# CD47 Enhances Cell Viability and Migration Ability but Inhibits Apoptosis in Endometrial Carcinoma Cells via the PI3K/Akt/mTOR Signaling Pathway

**DOI:** 10.3389/fonc.2020.01525

**Published:** 2020-08-26

**Authors:** Yun Liu, Yue Chang, Xinhong He, Yixuan Cai, Hao Jiang, Ru Jia, Jiyan Leng

**Affiliations:** Department of Obstetrics and Gynecology, Beijing Friendship Hospital Affiliated to Capital Medical University, Beijing, China

**Keywords:** CD47, endometrial carcinoma, cell viability, migration, apoptosis

## Abstract

**Purposes:** To measure expression levels of CD47 during endometrial carcinoma development, and to determine specific modulatory effects.

**Methods:** CD47 expression levels in endometrial carcinoma tissues and adjacent tissues were analyzed using qRT-PCR. CD47-overexpressed or downregulated cell models were established using CD47 plasmid or CD47 shRNA. The effects of CD47 on HEC-1A and Ishikawa cell growth were evaluated using CCK-8 assays. Migration ability of transfected HEC-1A and Ishikawa cells were examined using wound healing assays. Flow cytometry was used to measure the effects of CD47 on apoptosis and the cell cycle in HEC-1A and Ishikawa cells. Western blot was used to analyze the correlation between CD47 expression level and PI3K/Akt/mTOR signaling pathway.

**Results:** Highly expressed CD47 was observed in endometrial carcinoma tissues, with higher levels in more advanced tissues than in early tissues. Upregulation of CD47 enhanced cell viability and migration ability in HEC-1A and Ishikawa cells, while silencing CD47 caused the opposite results. CD47 overexpression suppressed apoptosis and inhibited cell cycle arrest in HEC-1A and Ishikawa cells. CD47 upregulation contributes to the activation of PI3K/Akt/mTOR signaling pathway in endometrial carcinoma cells.

**Conclusion:** CD47 exerts oncogenic functions in endometrial carcinoma by activating PI3K/Akt/mTOR signaling, suggesting it may be a novel immunotherapeutic target for therapeutic interventions.

## Introduction

Endometrial carcinoma accounts for ~85% of endometrial cancer; it is one of the commonly diagnosed gynecologic malignancies (1, 2). Despite the fact that many patients with endometrial carcinoma achieve good outcomes, mortality in advanced disease remains high (3). In recent years, treatments have not increased survival. For these reasons, clinically effective targets are needed.

Recently, immunotherapy has attracted increasing attention. Several studies demonstrated the ability of immunotherapy to improve the prognosis of various cancers via regulation of checkpoint inhibitors; it may also be effective in endometrial carcinoma (4). Piulats et al. reported two endometrial carcinoma patients in poor condition who were successfully treated with nivolumab, an anti-PD-1 monoclonal antibody (5). The patients were found to harbor mutations that may confer sensitivity to immune checkpoint inhibitors (5). Di et al. identified several regulatory targets of immunotherapy in endometrial carcinoma, suggesting new options for treatment (6). These findings suggest that immunotherapy is a promising therapeutic strategy to improve the prognosis of endometrial carcinoma patients.

CD47, the “don't eat me” signal, is an important immune checkpoint inhibitor that contributes to the progression of cancer (7). Normally, CD47 and its specific receptor, signal regulatory protein-α (SIRPα), are expressed in all cell types; however, they are upregulated in cancerous tissues and cell lines. Several studies have found that upregulation of CD47 promotes the progression of various tumors by blockading immune reactions (8, 9). For example, CD47 overexpression promoted the development of ovarian cancer by reducing the activity of macrophage phagocytosis (8). CD47 was upregulated in the membranes of human small-cell lung cancer cells, and Weiskopf et al. found that CD47 knockdown significantly suppressed tumor proliferation (10). CD47 also plays an oncogenic role in the progression of breast cancer (11). By synergistic action with inflammatory mediators, CD47 enhanced the migration capacity of colon tumor cells and promoted colon cancer development (12). Nevertheless, studies of CD47 in endometrial carcinoma are limited (13).

In recent years, the oncogenic effects of phosphatidylinositol 3-kinase (PI3K)/Akt/mammalian target of rapamycin (mTOR) signaling in cancer, including in endometrial carcinoma, have received increasing recognition (14, 15). Philip et al. reported that inhibition of PI3K/Akt/mTOR signaling increased the sensitivity of endometrial carcinoma cells to some targeted therapies (16). Cao et al. observed that claudin-6 (CLDN6) knockdown suppressed the proliferation and migration of endometrial carcinoma cell by regulating the PI3K/Akt/mTOR signaling pathway (17). A correlation between CD47 and the PI3K/Akt/mTOR pathway has also been identified. Liu et al. found that increased CD47 promoted human glioblastoma progression by modulating the PI3K/Akt signaling pathway (18). Nevertheless, there remain no studies of CD47-PI3K/Akt/mTOR signaling and endometrial carcinoma.

In the present study, we explored the relationship between CD47 expression and the development of endometrial carcinoma. We measured expression levels of CD47 in 22 paired carcinoma-normal endometrial tissues. CD47 overexpression- or knockdown-cell lines of endometrial carcinoma were established by transfection with plasmid or shRNA. Subsequently, functional experiments were performed to evaluate the effects of CD47 on endometrial carcinoma.

## Materials and Methods

### Tissue Collection

A total of 22 paired cancer-normal samples of endometrial carcinoma patients were collected from the Beijing Friendship Hospital Affiliated to Capital Medical University. These patients had not yet received chemoradiotherapy prior to sample collection. The pathological stages of endometrial cancer patients were classed according to the International Federation of Gynecology and Obstetrics (FIGO) criteria of endometrial cancer, including 10 stage III–IV and 12 stage I–II tumors. All samples were obtained during surgery and were stored for subsequent RNA analysis (Genstar, Beijing, China) at −20°C. Before tissue collection, written informed consent was obtained from the patients or their families. This study was approved by Ethics Review Board of Beijing Friendship Hospital Affiliated to Capital Medical University.

### Cell Culture

Human endometrial carcinoma cell lines, Ishikawa and HEC-1A, were obtained from Nanjing Keygen Biotech (Nanjing, China). Both cell types were maintained in Dulbecco's modified Eagle's medium (DMEM) (HyClone, Victoria, Australia), containing 1% penicillin/streptomycin and 10% fetal bovine serum (FBS) (Gibco, Gaithersburg, MD, USA). The two endometrial carcinoma cell lines were cultured in an incubator with a humidified atmosphere at 37°C and 5% CO_2_.

### Plasmid Construction and Transfection

Before transfection, HEC-1A and Ishikawa cells were seeded into 6-well plates at 1 × 10^5^ cells per well and cultured for 24 h. Subsequently, a total of 1 μg plasmid or shRNA was added to each well-plates to increase or reduce CD47 expression levels. pIRES2-ZsGreen1-CD47 (Hanbio, Shanghai, China) was used to up-regulate CD47 expression levels in HEC-1A and Ishikawa cells, while the empty plasmid was used as the control. The shRNA (5′-CCGGGCACAATTACTTGGACTAGTTCTCGAGAACTAGTCCAAGTAATTGTGCTTTTT-3′) that inhibits the expression of CD47, was purchased from GeneChem Biotech Company (Shanghai, China).

### qRT-PCR

Endometrial carcinoma tissues and the adjacent tissues were homogenized using a TissueLyser to isolate RNA. TRIzol regent (Invitrogen, Carlsbad, CA, USA) was used to extracted RNA from target tissues according to manufacturer's instructions. cDNA was synthesized using a reverse transcription kit (Takara, Dalian, China), and was then used to measure expression levels of CD47 using SYBR II Premix Taq (Takara, Dalian, China). The fold-changes of CD47 expression were calculated using the 2-ΔΔCT method, with GAPDH as the reference. The primers purchased from Sangon Biotech (Shanghai, China) were as follows:

CD47: Forward primer: 5′-AGAAGGTGAAACGATCATCGAGC-3′; Reverse primer: 5′-CTCATCCATACCACCGGATCT-3′.

GAPDH: Forward primer: 5′-ACCACAGTCCATGCCATCAC-3′; Reverse primer: 5′- TCCACCACCCTGTTGCTGTA-3′.

### Cell Viability Assay

HEC-1A and Ishikawa endometrial carcinoma cells were seeded into 96-well plates at 1 × 10^3^ cells per well, and were then transfected with CD47 plasmid or CD47 shRNA after incubation for 24 h. Subsequently, these cells were cultured at 37°C and 5% CO^2^ for 1 week to construct CD47 up-regulated or down-regulated cell lines. The viability of transfected cells was measured using a spectrophotometer after adding 10 μl CCK-8 (Dojindo, Kumamoto, Japan) into each well for 1 h. Absorbance (OD) at 450 nm was measured at 4 and 48 h after transfection, and the values at 4 h after transfection were used as the control level.

### Wound Healing Assay

To measure the effects of CD47 on endometrial carcinoma cell migration, a wound healing assay was performed. Briefly, 5 × 10^6^ target cells were transferred into 6-well plates and incubated at 37°C until 90% confluence. A 20 μl tip was used to scrape a straight wound in HEC-1A and Ishikawa endometrial carcinoma cell monolayers. After washing the cells with PBS, the plates were incubated for 24 h. Wound distance was measured under light microscopy.

### Flow Cytometry

After CD47 overexpressed or knockdown cell lines were established, apoptosis of targeted cells was measured by flow cytometry using an Annexin V-FITC/PI kit (GenStar, Beijing, China) according to the manufacturer's instructions. The cell cycle of transfected HEC-1A and Ishikawa cells were analyzed using the Cell Cycle and Apoptosis Analysis Kit (Beyotime Biotechnology, Shanghai, China), according to the manufacturer's protocol.

### Western Blot

Total protein was extracted from cells using radio immunoprecipitation assay lysis buffer (KeyGen BioTech, Nanjing, China) according to the manufacturer's instructions. Proteins were separated by gel electrophoresis with 12% sodium dodecyl sulfate polyacrylamide and transferred onto a polyvinylidene fluoride membrane (PVDF) (Millipore, Billerica, MA, USA). The membranes were then blocked in skimmed milk for 1 h at 20°C and then incubated with primary antibodies overnight at 4°C. Primary antibodies against PI3K (1:2000), Akt (1:2000), mTOR (1:2000), and glyceraldehyde-3-phosphate dehydrogenase (GAPDH) (1:500) were purchased from Cell Signaling Technology (Danvers, MA, USA). Membranes were cultured with secondary antibodies (Santa Cruz Biotechnology, Santa Cruz, CA, USA) the following day for 1 h at 20°C. The immunocomplexes were detected using an enhanced chemiluminescence detection kit (Thermo Fisher Scientific, Waltham, MA, USA).

### Statistical Analysis

Data were analyzed using SPSS version 19.0 software (IBM, Chicago, IL, USA) and *p* < 0.05 indicated statistical significance. All data were obtained by experiments at least in triplicate and were expressed as mean ± SD. The paired Student's *t*-test was used to determine significant differences between groups.

## Results

### CD47 Was Up-Regulated in Endometrial Carcinoma Tissues and Was Negatively Related to Pathological Stage

**Figure 1:**
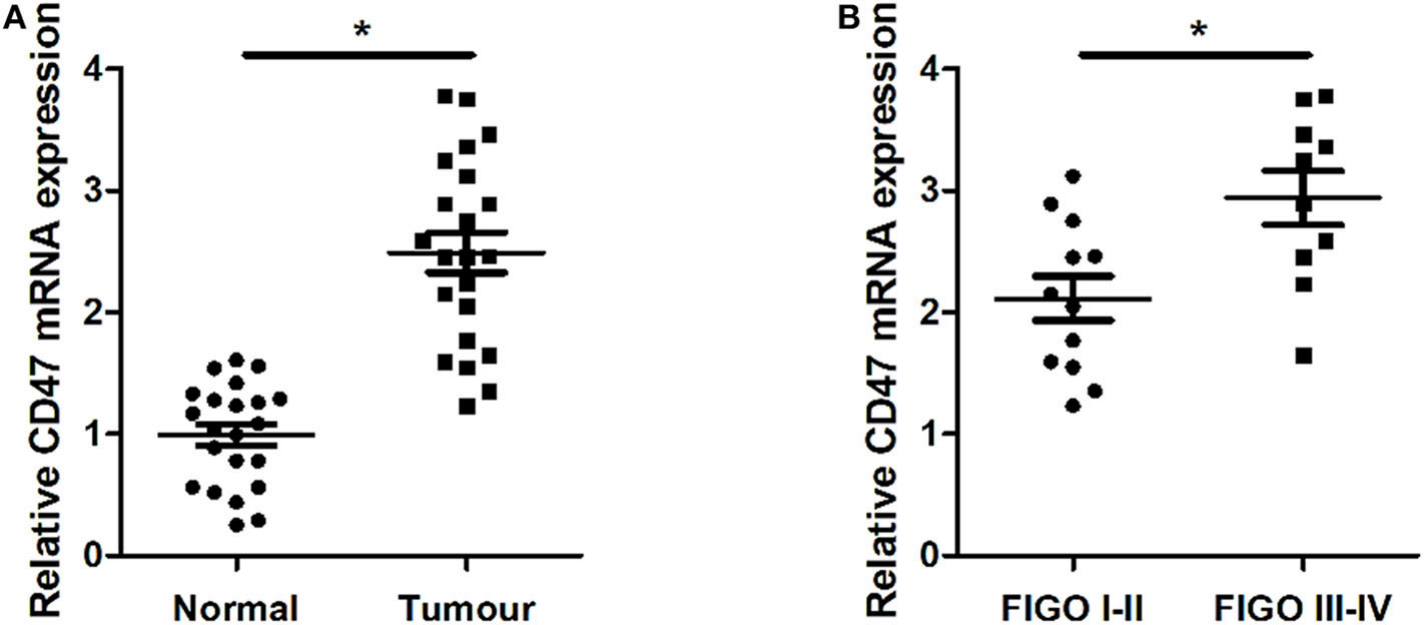
Up-regulation of CD47 mRNA expression levels measured in endometrial carcinoma tissues, and compared with adjacent tissues. **(A)** Expression levels of CD47 mRNA were relatively higher in endometrial carcinoma tissues. **(B)** Higher CD47 mRNA levels measured in endometrial carcinoma patients at advanced stages. *p < 0.05.

We analyzed expression levels of CD47 mRNA in two paired carcinoma tissues and adjacent normal tissues using qRT-PCR. We found that CD47 expression was upregulated in carcinoma tissues (Figure 1A). We classified pathological stages of endometrial cancer patients according to FIGO criteria and found CD47 mRNA expression levels were higher in the stage III-IV groups (Figure 1B), further suggesting a correlation between CD47 mRNA expression levels and endometrial carcinoma progression.

### CD47 Enhances the Cell Viability of HEC-1A and Ishikawa Endometrial Carcinoma Cells

**Figure 2:**
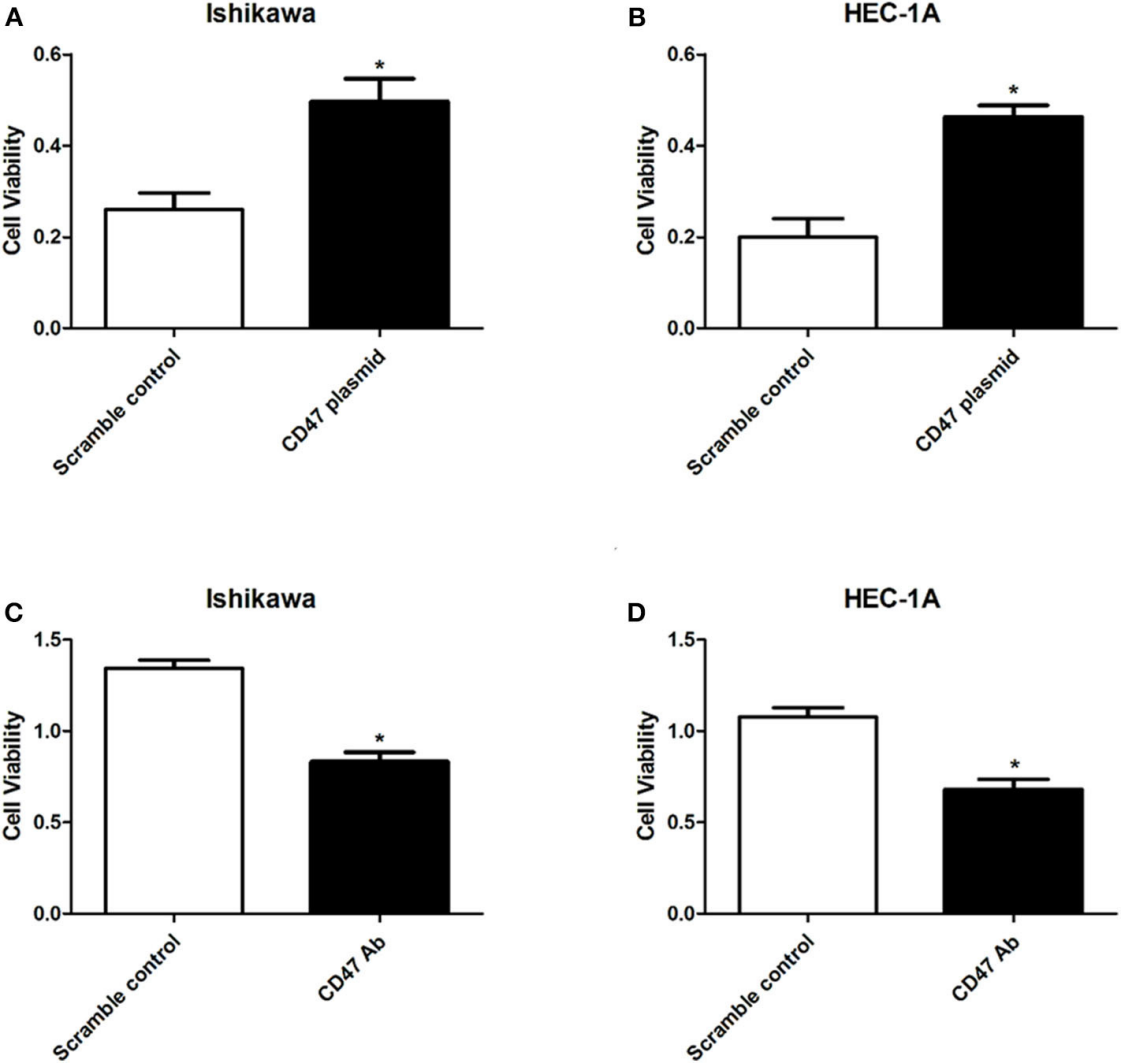
CD47 overexpression enhanced cell viability in HEC-1A and Ishikawa cells. **(A,B)** Overexpressed CD47 promoted cell proliferation in HEC-1A and Ishikawa cell lines. **(C,D)** CD47 knockdown inhibited cell proliferation in HEC-1A and Ishikawa cell lines. *p < 0.05.

To determine the regulatory effects of CD47 on endometrial carcinoma cell phenotypes, we selected endometrial carcinoma HEC-1A and Ishikawa cells for subsequent assays. CD47 plasmid, CD47 shRNA or the control were injected into HEC-1A and Ishikawa cells to construct CD47 up- or down-regulation cell models. Subsequently, the effects of CD47 on cell viability were measured using a CCK-8 assay. The results showed that up-regulation of CD47 promoted the proliferation of endometrial carcinoma cells (Figures 2A,B), while down-regulation of CD47 suppressed its growth (Figures 2C,D). Both results suggested that CD47 enhances cell viability in HEC-1A and Ishikawa cells.

### CD47 Promotes Cell Migration in HEC-1A and Ishikawa Endometrial Carcinoma Cells

**Figure 3:**
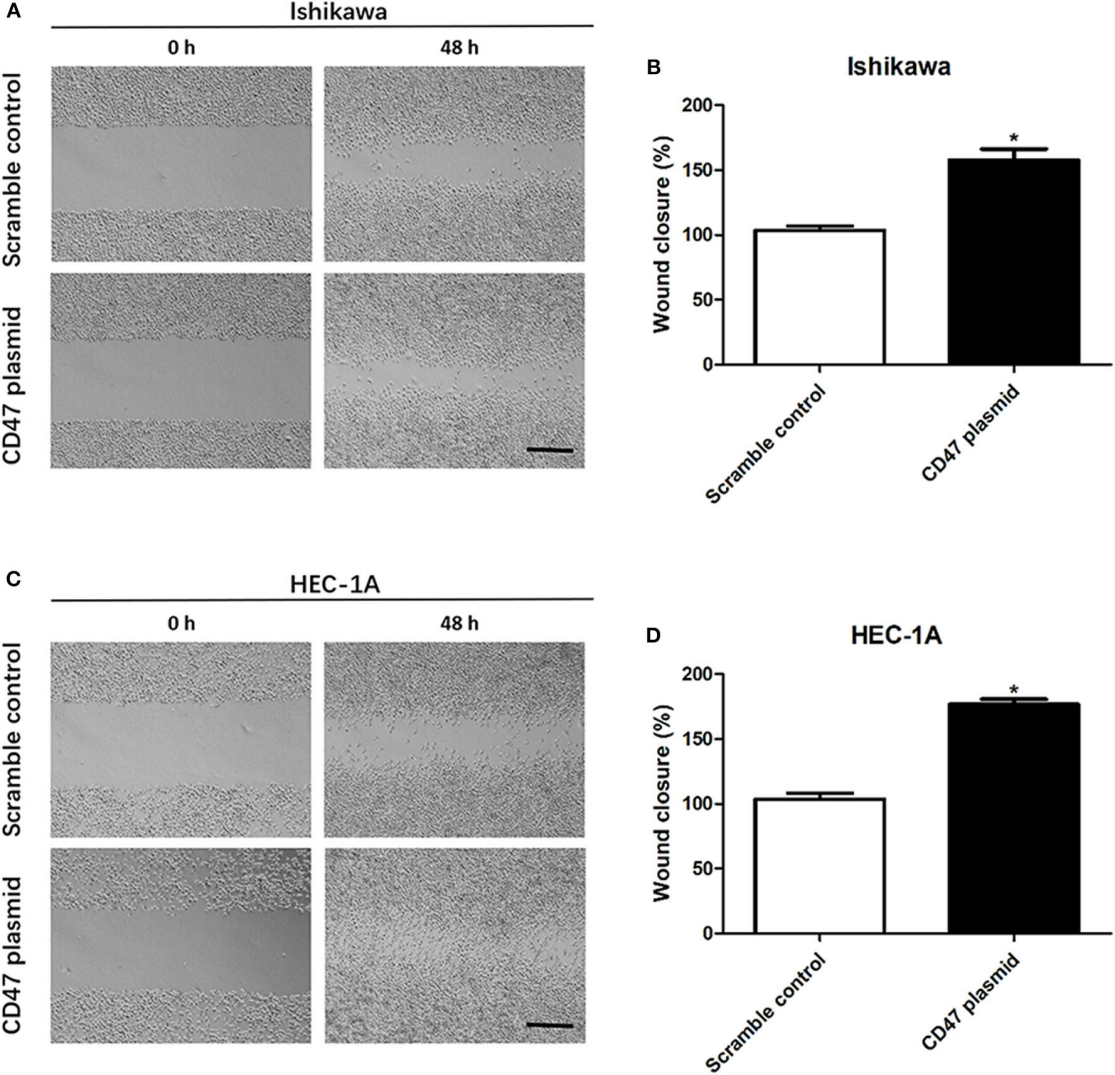
CD47 up-regulation promoted migration in HEC-1A and Ishikawa cells. **(A,B)** CD47 overexpression accelerated the rate of wound closure in Ishikawa cells. **(C,D)** CD47 overexpression accelerated the rate of wound closure in HEC-1A cells. Scale bar = 200 um. *p < 0.05.

To further determine the effects of CD47 on endometrial carcinoma progression, wound healing assays were performed to analyze the role of CD47 in endometrial carcinoma cell migration. The wound area was smaller in the CD47 overexpression group (Figures 3A–D), suggesting that increasing expression levels of CD47 contributed to migratory ability of HEC-1A and Ishikawa cells.

### Up-Regulation of CD47 Suppresses the Apoptosis and Cell Cycle Arrest of Endometrial Carcinoma Cells

**Figure 4:**
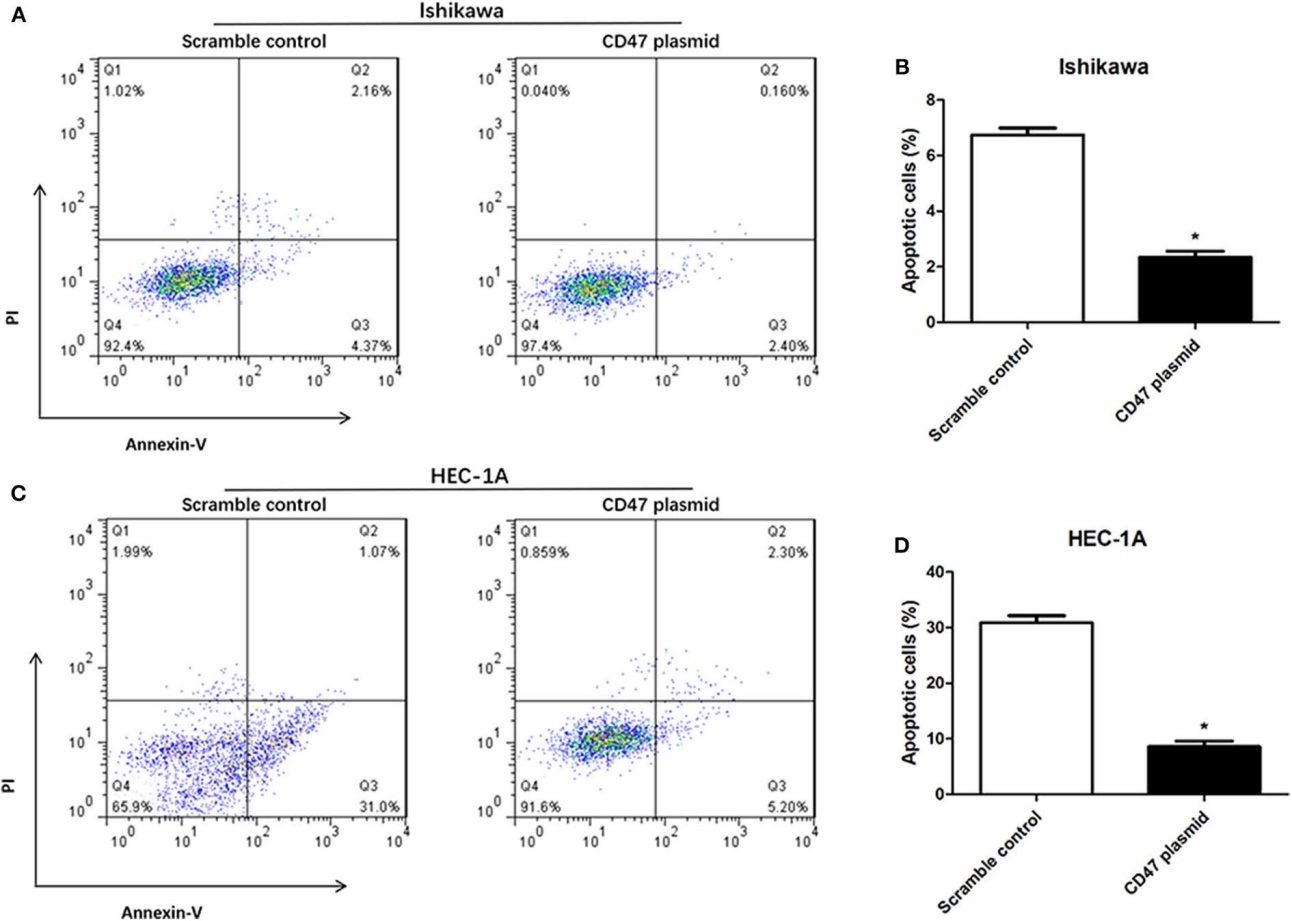
CD47 up-regulation inhibited apoptosis in HEC-1A and Ishikawa cells. **(A,B)** Up-regulation of CD47 inhibited cell apoptosis in Ishikawa cell line. **(C,D)** CD47 overexpression inhibited cell apoptosis in HEC-1A cell line. *p < 0.05.

**Figure 5:**
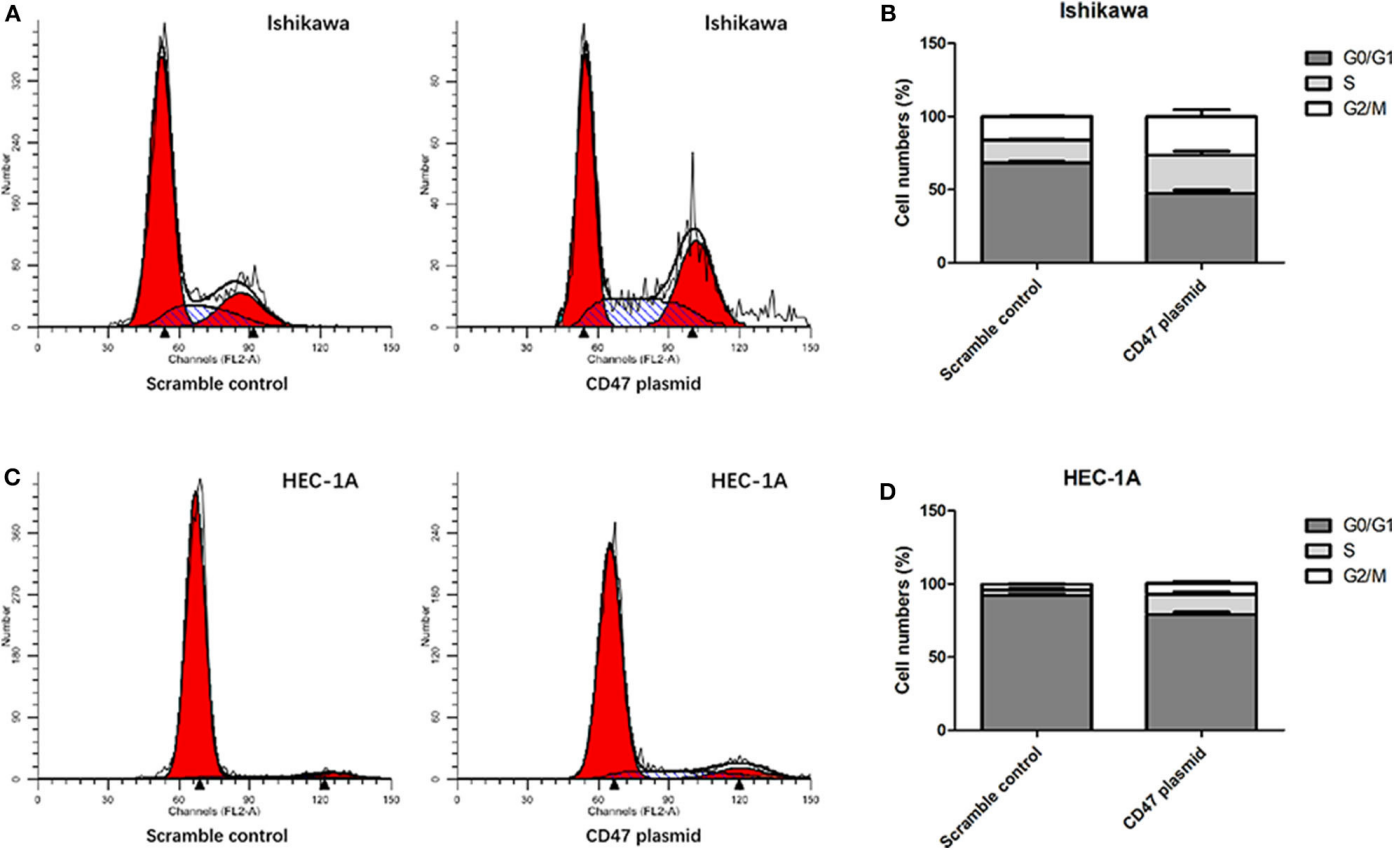
Up-regulation of CD47 promoted cell cycle arrested in HEC-1A and Ishikawa cells. **(A,B)** Upregulation of CD47 reduced the rate of S and G2.M stage cells in Ishikawa cell line. **(C,D)** Upregulation of CD47 reduced the rate of S and G2.M stage cells in HEC-1A cell line.

Flow cytometry assays were carried out to analyze the modulatory effects of CD47 on endometrial carcinoma cell cycle and apoptosis. The results showed that upregulating CD47 expression inhibited apoptosis in HEC-1A and Ishikawa cells (Figures 4A–D). We also detected a negative correlation between CD47 expression and the ratio of S and G2/M stage cells in HEC-1A and Ishikawa cells (Figures 5A–D), suggesting that up-regulation of CD47 inhibited apoptosis in endometrial carcinoma cells by modulating the cell cycle.

### CD47 Overexpression Activates PI3K/Akt/mTOR Signaling Pathway in Endometrial Carcinoma Cell Lines

**Figure 6:**
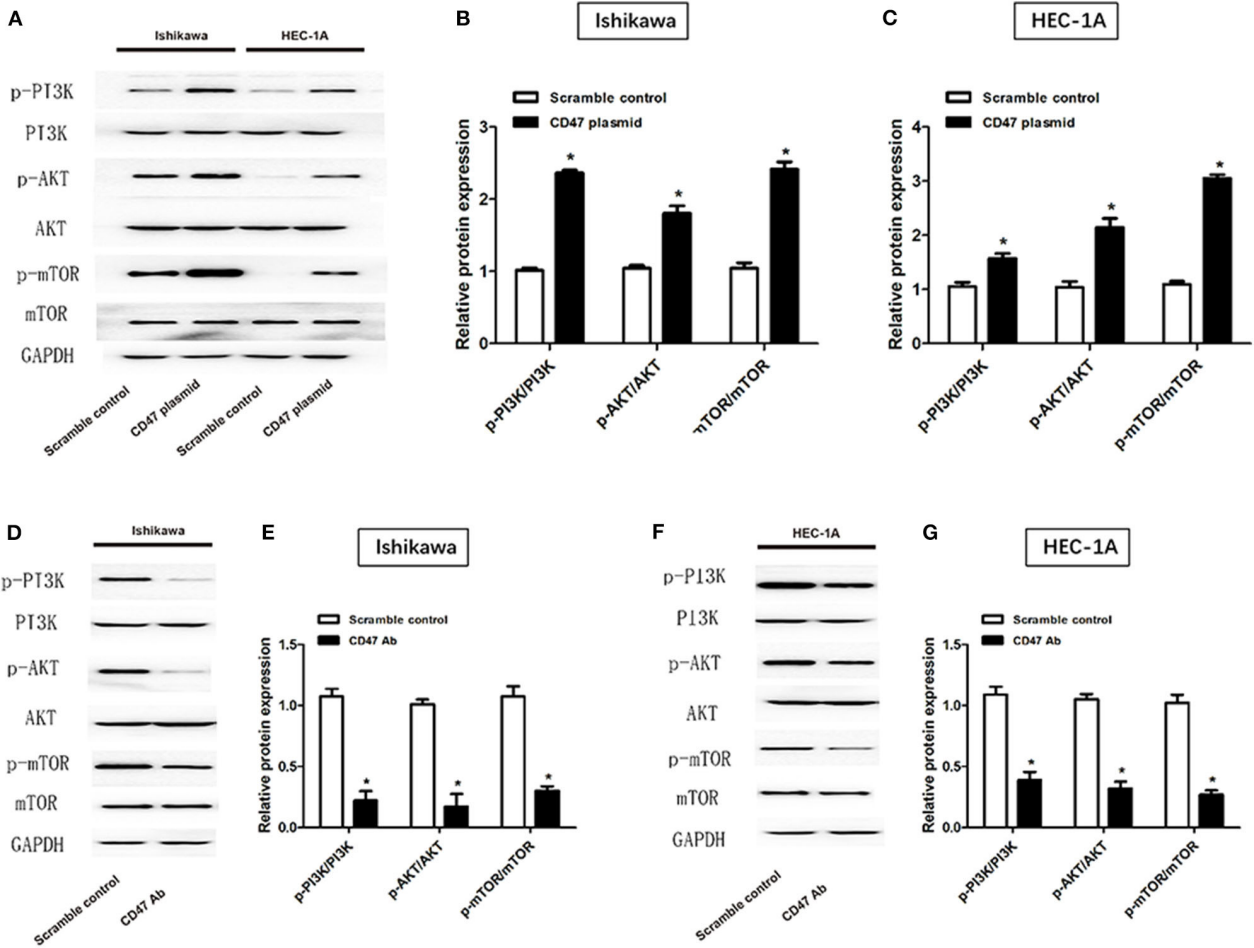
CD47 up-regulation activated PI3K/Akt/mTOR signaling pathway in HEC-1A and Ishikawa cell lines. **(A–C)** CD47 overexpression increased expression levels of PI3K, Akt, and mTOR proteins. **(D–G)** CD47 down-regulation decreased expression levels of PI3K, Akt, and mTOR proteins. *p < 0.05.

To investigate the mechanisms underlying the effects of CD47 on endometrial carcinoma, western blot was performed to measure PI3K/Akt/mTOR signaling pathway protein levels after injection of CD47 plasmid into endometrial carcinoma cells. Expression levels of PI3K, Akt and mTOR were measured in CD47 plasmid injected Ishikawa and HEC-1A cells. We found that CD47 up-regulation increased PI3K, Akt and mTOR expression in endometrial carcinoma cells (Figures 6A–C), suggesting that CD47 exerted oncogenic effects in endometrial carcinoma via up-regulating the PI3K/Akt/mTOR signaling pathway. Besides, CD47 down-regulation reduced PI3K, Akt, and mTOR expression in endometrial carcinoma cells (Figures 6D–G) further confirmed this.

## Discussion

The incidence of endometrial cancer is only less than that of cervical cancer and ovarian cancer among gynecologic malignancies and is rapidly increasing (19). Though the prognosis of many endometrial carcinomas is good, there are frequent relapses and metastases in advanced patients, giving rise to low survival rates (20). In recent decades, surgical treatment has not improved the survival of advanced endometrial cancer patients, suggesting that new approaches are needed.

In this present study, we measured expression levels and regulatory effects of CD47 in endometrial carcinoma. CD47 was upregulated in endometrial carcinoma tissues compared with normal endometrial tissue, and higher CD47 expression was observed in endometrial carcinoma patients with later pathological stage, compared with the early stage. Both CD47 expression assays revealed that CD47 was positively associated with endometrial carcinoma development. After transfected cells were established, functional experiments were performed to analyze the modulatory effects of CD47 on endometrial carcinoma cells. We found that up-regulation of CD47 promoted the proliferation and migration of HEC-1A and Ishikawa endometrial carcinoma cells, and inhibited apoptosis. CD47 down-regulation was a significant suppressive factor of endometrial carcinoma cell viability. All these results reconfirmed that CD47 is an important oncogenic factor in endometrial carcinoma.

Recently, several studies found that immunotherapy may be an effective strategy in the treatment of endometrial carcinoma (21). Generally, the microenvironment of tumor contributes to the immune evasion of cancerous cells by expressing proteins such as programmed cell death-1 (PD-1) and B7-H4 (22, 23). It may be the case that expression of these proteins may change the activity of immune system in cancer cells. For example, Sobecki et al. reported that anti-PD-1 immunotherapy may be an appropriate treatment for endometrial cancer patients with mismatch repair-deficiencies (24). There also are other checkpoint inhibitors that can act as immunotherapeutic targets in endometrial carcinoma (25). The CD47/SIRPα axis was also identified as an immune checkpoint inhibitor in cancer therapy (9, 26). A previous study observed that CD47 suppresses the progression of endometrial cancer cells by enhancing the sensitivity of M2 Polarized Macrophages on tumor cells (13). In this study, CD47 overexpression was also found a protective mechanism in endometrial carcinoma development and down-regulation of CD47 inhibited the proliferation and migration of endometrial carcinoma cells. Blocking CD47 may be an immunotherapeutic pathway for the treatment of endometrial carcinoma.

After binding with the growth factor ligand, membranous receptor tyrosine kinases and G protein-coupled receptors are activated, thereby stimulating the production of phosphatidylinositol- 3,4,5-trisphosphate (PIP3), originated from PI3K (27). Subsequently, PIP3 interacts with Akt, thereby increasing expression levels of mTOR (28–30). Up-regulation of the PI3K/Akt/mTOR signaling cascade causes disturbance of cell survival and growth, ultimately resulting in a competitive proliferation advantage, angiogenesis, metastatic competence, and therapeutic resistance. In this manner, the signaling pathway is a potential target for the treatment of various cancers (31, 32). We observed overactivity of the PI3K/Akt/mTOR signaling pathway in CD47 overexpressing Ishikawa and HEC-1A cell lines. These results suggest that CD47 promotes endometrial carcinoma progression via regulating the PI3K/Akt/mTOR signaling pathway.

## Conclusion

We found that CD47 expression was higher in endometrial carcinoma tissues, compared with the adjacent tissues. Upregulation of CD47 promoted progression of endometrial carcinoma by activating PI3K/Akt/mTOR signaling pathway. These findings suggest a novel immunotherapeutic pathway for investigating interventions to treat endometrial carcinoma.

## Data Availability Statement

The original contributions presented in the study are included in the article/supplementary files, further inquiries can be directed to the corresponding author/s.

## Ethics Statement

The studies involving human participants were reviewed and approved by Beijing Friendship Hospital Affiliated to Capital Medical University. The patients/participants provided their written informed consent to participate in this study.

## Author Contributions

YL designed the study, wrote the paper, and had overall responsibility for the research. YL, YCh, XH, HJ, RJ, JL, and YCa carried out the experiments. YCh and YCa contributed to data analysis. YL, XH, HJ, RJ, and JL revised the first-manuscript and submitted revised-manuscript. All authors approved the final manuscript version. All authors contributed to the article and approved the submitted version.

## Conflict of Interest

The authors declare that the research was conducted in the absence of any commercial or financial relationships that could be construed as a potential conflict of interest.
